# Social relationships and survival in the older adult cohort*

**DOI:** 10.1590/1518-8345.3844.3395

**Published:** 2021-01-08

**Authors:** Mariangela Uhlmann Soares, Luiz Augusto Facchini, Fúlvio Borges Nedel, Louriele Soares Wachs, Marciane Kessler, Elaine Thumé

**Affiliations:** 1 Universidade Federal de Pelotas, Pelotas, RS, Brazil.; 2 Scholarship holder at the Coordenação de Aperfeiçoamento de Pessoal de Nível Superior (CAPES), Brazil.; 3 Universidade Federal de Santa Catarina, Centro de Ciências da Saúde, Florianópolis, SC, Brazil.

**Keywords:** Aged, Social Support, Mortality, Longitudinal Studies, Health of the Elderly, Interpersonal Relations, Idoso, Apoio Social, Mortalidade, Estudos Longitudinais, Saúde do Idoso, Relações Interpessoais, Anciano, Apoyo Social, Mortalidad, Estudios Longitudinales, Salud del Anciano, Relaciones Interpersonales

## Abstract

**Objective:**

to verify the influence of social relations on the survival of older adults living in southern Brazil.

**Method:**

a cohort study (2008 and 2016/17), conducted with 1,593 individuals aged 60 years old or over, in individual interviews. The outcomes of social relations and survival were verified by Multiple Correspondence Analysis, which guided the proposal of an explanatory matrix for social relations, the analysis of survival by Kaplan-Meier, and the multivariate analysis by Cox regression to verify the association between the independent variables.

**Results:**

follow-up was carried out with 82.5% (n=1,314), with 46.1% being followed up in 2016/17 (n=735) and 579 deaths (36.4%). The older adults who went out of their homes daily had a 39% reduction in mortality, and going to parties kept the protective effect of 17% for survival. The lower risk of death for women is modified when the older adults live in households with two or more people, in this case women have an 89% higher risk of death than men.

**Conclusion:**

strengthened social relationships play a mediating role in survival. The findings made it possible to verify the importance of going out of the house as a marker of protection for survival.

## Abstract (pt)

**Objetivo:**

verificar a influência das relações sociais na sobrevivência de idosos residentes no sul do Brasil.

**Método:**

estudo de coorte (2008 e 2016/17), realizado com 1.593 indivíduos com 60 anos ou mais, em entrevistas individuais. Os desfechos relações sociais e sobrevivência foram verificados por Análise de Correspondências Múltiplas que orientou a proposição de matriz explanatória para relações sociais e a análise de sobrevivência por Kaplan-Meier e análise multivariada por regressão de Cox para verificar a associação entre as variáveis independentes.

**Resultados:**

o acompanhamento foi realizado com 82,5% (n=1.314), sendo 46,1% acompanhados em 2016/17 (n=735) e 579 óbitos (36,4%). O idoso que saiu de casa diariamente teve uma redução de 39% na mortalidade e ir a festas manteve o efeito protetor de 17% para sobrevivência. O menor risco de morte para as mulheres é modificado quando os idosos vivem em domicílios com duas ou mais pessoas, neste caso as mulheres apresentam risco 89% maior de morte do que os homens.

**Conclusão:**

relações sociais fortalecidas exercem papel mediador na sobrevivência. Os achados permitiram verificar a importância de sair de casa como marcador de proteção para a sobrevivência.

## Abstract (es)

**Objetivo:**

verificar la influencia de las relaciones sociales en la supervivencia de adultos mayores residentes en el sur de Brasil.

**Método:**

estudio de cohorte (2008 y 2016/17), realizado con 1.593 individuos de 60 años o más, en entrevistas individuales. Los resultados de las relaciones sociales y la supervivencia se verificaron mediante Análisis de Correspondencias Múltiples que guio la proposición de una matriz explicativa de las relaciones sociales y el análisis de supervivencia por Kaplan-Meier y el análisis multivariado por regresión de Cox para verificar la asociación entre variables independientes.

**Resultados:**

el seguimiento se llevó a cabo con el 82,5% (n=1.314), siendo que en 46,1% el seguimiento se practicó entre 2016/17 (n=735) y se registraron 579 óbitos (36,4%). El adulto mayor que salió de casa a diario tuvo una reducción del 39% en la mortalidad y el hecho de ir a fiestas mantuvo el efecto protector del 17% en la supervivencia. El menor riesgo de muerte para las mujeres se modifica cuando los adultos mayores viven en domicilios con dos o más personas, en este caso las mujeres presentan un riesgo de muerte 89% más alto que los hombres.

**Conclusión:**

el fortalecimiento de las relaciones sociales ejerce un papel mediador en la supervivencia. Los hallazgos permitieron comprobar la importancia de salir de casa como marcador de protección en la supervivencia.

## Introduction

Social relationships are interactions established by individuals throughout life, resulting from the broad set of political, economic, educational, occupational, cultural, and family systems. These social interactions promote the exchange of feelings capable of enhancing or mitigating the offer and receipt of assistance related to health maintenance^(^ 1 ^-^ 3 ^)^. The studies on social relationships use different terminologies to express conceptions, approaches, and cuttings of the theme, with emphasis on social support, networks, integration, and ties^(^ 1 ^-^ 2 ^,^ 4 ^-^ 6 ^)^.

Since Durkheim, at the end of the 19^th^ century, the effect of social relations on survival has been object of study^(^ 7 ^)^. There is diverse evidence of the need to understand the social interactions for the older adults, in high-income countries, starting in the 1950s of the 20^th^ century, with the emergence of classic theories about aging, due to their demographic relevance^(^ 2 ^)^.

The understanding of the influence of formal and informal social relationships on health conditions and mortality can be detailed by examining their structure and function aspects^(^ 2 ^,^ 8 ^-^ 9 ^)^. The structure assesses the quantity and type of relationships established, in the formal sphere, such as at work, in the use of the health services, and in participation in religious and cultural groups. In the informal sphere, the structure assesses the number and type of relationships in the family, such as with the spouse, children, and residents in the home. The function expresses the quality of the social relationships (formal and informal): whether positive or negative, whether satisfactory or conflicting, whether supportive or stressful^(^ 8 ^)^.

Diverse evidence signal the importance of relationships for quality of life in aging, being positive when frequent meetings take place and instrumental and emotional support is received^(^ 4 ^,^ 10 ^)^. Longitudinal studies^(^ 7 ^)^ verified an increase in mortality among the older adults with little diversity or low frequency of contact with other people and decreasing levels of social integration (measured by a synthetic indicator that reflects ties with the spouse, close friends, and relatives and participation in religious and other types of groups).

In the older adult population, the risk of death is higher among men with less perceived social support^(^ 11 ^)^; older adults who lived with other people had a lower risk of death than those who lived alone^(^ 12 ^)^. Locomotion difficulties, older age, being male, poor self-rated health, pre-frailty or frailty were considered predictors of mortality among the older adults in studies conducted in Brazil^(^ 13 ^)^. In contrast, strengthening the social relationships can also minimize the effect of socioeconomic conditions on the health situation and mortality of the most vulnerable older adults^(^ 6 ^)^.

In the last 30 years there has been greater systematization of the theoretical and empirical bases of the causal impact of social relationships on health^(^ 9 ^,^ 14 ^)^. However, the mechanisms by which social relationships affect health have yet to be explored^(^ 5 ^,^ 10 ^,^ 15 ^)^. In this sense, this study verified the influence of social relationships on the survival of older adults living in southern Brazil, with a special interest in identifying indicators capable of explaining the effect of the structure of formal and informal relationships.

## Method

This is a prospective cohort established between July and November 2008^(^ 16 ^)^, with a representative sample of individuals aged 60 years old or over living in the urban area of the municipality of Bagé. Between September 2016 and August 2017, a follow-up study was conducted with the location of the 1,593 identified at the beginning of the cohort.

The municipality of Bagé is located in the state of Rio Grande do Sul (RS), in the extreme south of Brazil. In the 2010 census it recorded a population of 116,794,000 inhabitants, 14.7% of the population aged 60 or over, a demographic density of 28.52 inhabitants/km² and 86% of the population living in the urban area, with a Municipal Human Development Index (MHDI) of 0.740 in 2010, considered high.

The selection of the sample, in 2008, occurred based on the delimitation of the areas covered by all the fifteen primary health care services in the urban area, selecting with a systematic jump one in six households and interviewing all the older residents^(^ 16 ^)^. Data collection for the monitoring occurred from the visit to the homes and the search of the participating residents. Those who changed residence were located in the new public places.

As it is a population-based study with residents in the community, in the constitution of the cohort, institutionalized older adults or those deprived of their liberty were excluded and, in the follow-up, the participants who passed to this condition were designated as losses. In both periods of data collection, the individuals who were not at their homes after three attempts were considered losses. Disabled older adults were interviewed with the help of a companion, with questions of self-perceived health not being applied to those who were unable to answer alone.

The first data collection used a printed questionnaire, with subsequent coding and double typing to form the database. The follow-up made use of the Personal Digital Assistant (PDA) electronic device, with questions previously coded, and digitally transferred to the database.

The logistics to identify the deaths that occurred in the period included the nominal search in the Mortality Information System (*Sistema de Informação de Mortalidade*, SIM) together with the 7^th^ Regional Health Coordination of RS, and with the health surveillance nucleus of the State Health Secretariat of RS in 2018, in the homes proper during the home visits and in the civil registry offices of the municipalities where the death occurred when informed by the family member.

The dependent variables represent the structure of the formal and informal social relations, based on the theoretical conception previously proposed^(^ 8 ^)^, with investigation of the following variables: marital status (single or without a partner/married or with a partner/widower); number of residents in the home (lives alone/older adult and one person/older adult and two or more people); receiving and offering financial assistance, housing and companionship or personal care between the older adult and the family (yes/no); if they went out of the house in a typical week in the last thirty days (yes/no/frequency); visiting family and friends (yes/no/frequency); leisure activities in the last 30 days (yes/no); going to mass (yes/no), participating in dancing activities (yes/no), family or community party (yes/no), having traveled to another city or going on a tour (yes/no), participating in a workshop or group (yes/no), and having attended a funeral or burial (yes/no).

The Multiple Correspondence Analysis (MCA)^(^ 17 ^)^ guided the selection and grouping of variables and categories for the proposal of an explanatory matrix with less variability, with no harm to the grouped information. The MCA has shown that “going out of the house”, “going to parties” and “number of residents in the home”, are indicators of the social relationships.

The independent variables were the following: gender (male/female); years of study (illiterate/from 1 to 7 years/8 or more years); socioeconomic classification of the Brazilian Association of Research Companies (*Associação Brasileira de Empresas de Pesquisa*, ABEP) (A/B/C/D/E); smoking (no/yes); functional difficulty to perform basic activities of daily living (BADLs); needing help to perform one or more of the self-care activities: eating, bathing, grooming, dressing up, mobilizing, maintaining control over their eliminations^(^ 18 ^)^ (Without difficulty/With difficulty); and multimorbidity - occurrence of two or more morbidities, which are: depression - Geriatric Depression Scale – GDS^(^ 19 ^)^, cognitive deficit - Mini-mental State^(^ 20 ^)^, high systemic blood pressure, diabetes mellitus, spinal problems, and rheumatic diseases - self-reported medical diagnosis (yes/no).

In the first analysis, an estimate of the median age of death was calculated, calculated by the Kaplan-Meier method, and subsequent point and interval analysis of the mortality rate, calculated by the Poisson distribution, for the global sample and for each independent variable analyzed.

The survival analysis (time elapsed until death occurred) was performed using the Cox model, taking as the initial observation time the age in days (date of the first interview - date of birth) and as final time, the age + the observation time (time at risk) until the event occurred (death or censorship). This method, called the counting process, was preferred for giving meaning to the survival time, which is no longer simply a count of days and becomes the age of the person^(^ 21 ^)^. Thus, all the covariates have their values (effect and significance) adjusted for age, which appears as the time scale to the left of the equation, in the following form:

*age at the time of the event ≃ effect of the covariates + random variability*

The analysis model was built by adding variables from the null model, starting with the social relationships indicator variables (going out of the house, going to parties, and the number of residents in the home), followed by the biological based variables, but closely related to the culture, including gender and functional difficulty for activities of daily living and ending with indicators of the social condition of the older adult, including schooling and socioeconomic classification.

Four possible interactions were tested: the first between smoking and multimorbidity, due to the fact that smoking is considered a risk factor for chronic non-communicable diseases and mortality^(^ 11 ^,^ 22 ^)^; the second between schooling and socioeconomic classification, because the ways of organizing social groups are built as a survival strategy for individuals and can protect the poorest and least educated^(^ 1 ^,^ 6 ^,^ 14 ^,^ 23 ^)^; the third tested interaction was between functional difficulty and going out of the house, in order to verify the effect on mortality of limitations for activities of daily living and of the difficulty in going out of the house^(^ 24 ^-^ 25 ^)^; and finally, the interaction between gender and the number of residents in the home, justified by the gender relations at this age, in which the social role of women as caregivers can be intensified^(^ 8 ^)^. The final model selected was considered to be a good fit as it did not refute the assumption of proportionality for the hazards, being significantly different from the null model and not different from the saturated model, at the 5% level.

The study was approved in its ethical aspects by the Research Ethics Committee of the Medical School of the Federal University of Pelotas, under registries No. 15/08, in 2008, and No. 678,664, in 2014.

## Results

82.5% (n=1,314) of the 1,593 older adults interviewed in 2008 were found. Of this total, 46.1% were interviewed in 2016/17 (n=735) and 579 deaths were identified (36.4%). The losses represented 12.4% (n=198), and 53 of them occurred due to change of city of residence, seven due to institutionalization, 59 due to deaths without confirmation of date, 22 due to problems in digital data transfer, and 57 older adults were not found. The refusals (n=81) represented 5.1% of the respondents in 2008, and 6.1% of those located in this study.

Of the 1,314 older adults, 25% died before reaching 70 years of age, half before the age of 78.0 years old, and the rest were still alive and over 85 years old. After 8.1 years of follow-up, the median age was 78 years old.

During the follow-up period, practically half of the men and a third of the women died, with a difference in the median age between men and women of three years and five months old, with the mortality rate of men significantly higher than that of women. The difference in the median between illiterates and those with 8 or more years of study was 2 years and 3 months. Low schooling significantly increased the mortality rate. At the end of the follow-up, the median age of death of the older adults belonging to the A/B (richest) economic classification was almost three years higher than that of the D/E (poorest) categories; the same occurred with the mortality rate of the poorest older adults being significantly higher than that of the richest. The older adults who, at the beginning of the follow-up, reported smoking and those who had multimorbidities, had a shorter survival time than those who did not smoke or did not have multimorbidities, but the difference in the rates was not statistically significant. The mortality rate for the older adults who live with more people is higher when compared to the other categories (Table 1).

**Table 1:** Distribution of the number of individuals followed-up, number of deaths, median age at the end of the observation study in years, and death rates per thousand individuals-year. SIGa-Bagé Study, Bagé, RS, Brazil, 2008 - 2016/17

	n	Deaths	Age*	Rate (‰)	95CI^†^	
Global						
Global	1,314	579	77y11m	67.56	62.05	73.06
Gender						
Male	499	245	76y2m	78.14	68.35	87.92
Female	815	334	79y7m	61.45	54.86	68.04
Schooling						
Illiterate	311	158	76y2m	81.41	68.72	94.10
1 to 7 years	732	319	77y11m	66.83	59.49	74.16
8 to 20 years	270	101	78y5m	54.48	43.85	65.10
Economic classification						
A/B	340	122	79y2m	52.97	43.57	62.37
C	506	229	78y4m	70.56	61.42	79.70
D/E	458	220	76y3m	73.97	64.19	83.74
Smoking						
No	1,110	482	78y10m	66.33	60.41	72.25
Yes	204	97	74y6m	74.40	59.60	89.21
Multimorbidity						
No	569	235	78y1m	62.41	54.43	70.38
Yes	745	344	76y11m	71.59	64.03	79.16
Functional difficulty						
No difficulty	1,164	457	78y7m	57.54	52.26	62.81
With difficulty	150	122	68at9m	194.38	159.89	228.87
Number of residents in the home						
Alone	229	103	77y11m	67.54	54.5	80.59
Older adult and 1 other person	434	183	78y7m	63.62	54.4	72.84
Older adult and 2 or more people	649	291	77y8m	70.05	62.0	78.10
Going out of the house						
Not at all	202	138	71y6m	137.67	114.70	160.64
Once a week	292	139	75y7m	75.34	62.82	87.87
2 to 4 times/week	352	145	77y9m	60.13	50.35	69.92
Every day	468	157	80y8m	47.40	39.99	54.82
Going to parties						
No	904	444	76y3m	78.09	70.83	85.35
Yes	410	135	80y10m	46.80	38.90	54.69

* Age = Median age at the end of the follow-up, calculated by the Kaplan-Meier method;

† 95CI = 95% Confidence interval, calculated by Poisson distribution

The survival of older adults without functional difficulties and of those who went out of their houses every day of the week at the beginning of the follow-up was about a decade longer than that of those with functional difficulties and of those who did not go out of their homes at all. The mortality rate was practically three times lower among those who went out of their houses every day and among those who did not have functional difficulties. The median age of death for the older adults who, at the beginning of the follow-up, reported going to parties was nearly four years higher than that of those who answered negatively to this question, with a significant difference in the mortality rate (Table 1).

**Figure 1:**
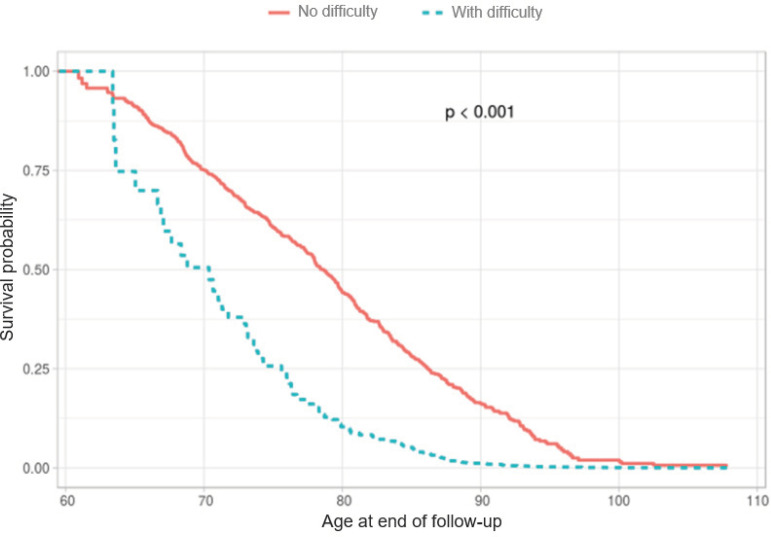
Probability of survival according to the presence of functional difficulties for basic activities of daily living. SIGa-Bagé Study, 2008-2016/17. Pelotas, RS, Brazil, 2019

Figure 1 illustrates the survival curves among the individuals according to the functional difficulty for basic activities of daily living, at the beginning of the follow-up. At 80 years old, practically half of the older adults with preserved functional difficulties were still alive, while the proportion of those with functional difficulties was approximately 12.5% (Figure 1).

**Figure 2:**
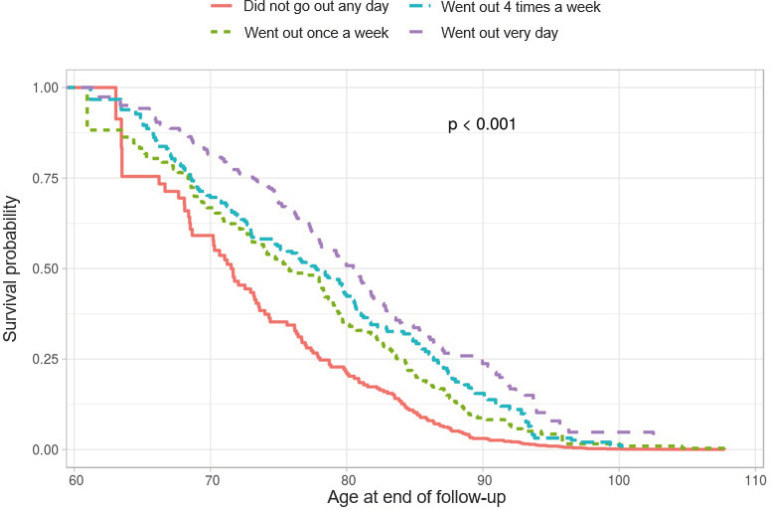
Probability for survival according to social relationships (going out of the house during the week). SIGa-Bagé Study, 2008-2016/17. Pelotas, RS, Brazil, 2019

In the gross analysis, the probability of survival grows due to the increase in the number of days that the older adults go out of their houses during the week. At 80 years old, almost half of the older adults who went out of their houses every day of the week were still alive, while the proportion of those who did not go out any day was approximately 25%. At the age of 90, there were practically no more older adult survivors who, at the beginning of the study, reported not going out of their houses any day of the week (Figure 2).

In the adjusted analysis, the risk of death was 57% lower for women, compared to men (p<0.001). However, this effect was modified by the number of residents in the home (p=0.03), and it was observed that, among older adults living in households with more than two people, women have an 89% higher risk for death than men (p=0.01) (Table 2).

**Table 2:** Probability of death according to the independent variables (n=579). SIGa-Bagé Study, Bagé, RS, Brazil, 2008 - 2016/17

Risk factors	Gross Analysis			Adjusted Analysis		
	HR (CI_95_)	p.Wald*	p.LLH^†^	HR (CI_95_)	p.Wald*	p.LLH^†^
Gender			<0.001			<0.001
Male	1	--		1	--	
Female	0.68 (0.58;0.81)	<0.001		0,43 (0,29; 0,64)	<0.001	
Schooling						
8 years or more	1	--		1	--	
1 to 7 years	1,14 (0,91; 1,42)	0.262		1,03 (0,69; 1,52)	0.90	
Illiterate	1,02 (0,79; 1,32)	0,885		2,12 (1,22; 3,68)	0.007	
Economic classification			0.097			0.20
A/B	1	--		1	--	
C	1.27 (1.02; 1.58)	0.036		1.85 (1.21; 2.83)	0.004	
D/E	1.21 (0.96; 1.51)	0.102		1.33 (0.56; 3.11)	0.51	
Smoking			<0.001			<0.001
No	1	--		1	--	
Yes	1.57 (1.25; 1.96)	<0.001		1.48 (1.17; 1.86)	0.001	
Multimorbidity			0.304			--
No	1	--		--	--	
Yes	1.09 (0.92; 1.29)	0.305		--	--	
Functional difficulty	<0.001					<0.001
No difficulty	1	--		1	--	
With difficulty	2.18 (1.75;2.72)	<0.001		1.95 (1.52; 2.50)	<0.001	
Number of residents in the home			0.179			0.65
Alone	1	--		1	--	
Older adult and 1 other person	0.96 (0.75; 1.22)	0.745		0.76 (0.52; 1.10)	0.14	
Older adult and 2 or more people	1.14 (0.91; 1.43)	0.268		0.65 (0.45; 0.93)	0.02	
Going to parties			<0.001			0.02
No	1	--		1	--	
Yes	0.68 (0.56; 0.83)	<0.001		0.83 (0.67; 1.02)	0.08	
Going out of the house			<0.001			<0.001
Not at all	1	--		1	--	
Once a week	0.69 (0.54; 0.88)	0.002		0.77 (0.59; 1.00)	0.05	
2 to 4 times/week	0.61 (0.48; 0.78)	<0.001		0.78 (0.60; 1.02)	0.07	
Every day	0.48 (0.38; 0.61)	<0.001		0.61 (0.46; 0.80)	<0.001	
Interactions						
Gender and number of residents						0.03
Older adult and 1 other person: Female	--	--	--	1.39 (0.85; 2.28)	0.19	
Older adult and 2 or more people: Female	--	--	--	1.89 (1.18; 3.03)	0.008	
Schooling and economic classification						0.004
1 to 7 years: C	--	--	--	0.70 (0.41; 1.19)	0.19	
Illiterate: C	--	--	--	0.25 (0.12; 0.50)	<0.001	
1 to 7 years: D/E	--	--	--	1.04 (0.42; 2.58)	0.94	
Illiterate: D/E	--	--	--	0.36 (0.13; 0.96)	0.04	

* Tests the null hypothesis that the hazard for death is the same in all the categories of the variable;

† Tests the null hypothesis that the global hazard for death does not change because of the variable. Model information: n: 1,301; No. of events: 568; Likelihood ratio test: 119.2 in 18 DoF, p<0.001; AIC: 5898.7

Although schooling and economic classification alone were not associated with survival at the 5% level, there is an interaction between the effects of both on survival. Thus, inferences cannot be made about the isolated effect of each variable, but about their interaction: among the older adults belonging to the C and/or D/E categories, being illiterate was a protective factor, reducing the risk for mortality by 75% and 64%, respectively, compared to those with higher schooling (Table 2).

In the adjusted model, among smokers the risk of death was 48% higher than that observed in non-smokers (p<0.001). Among the older adults with functional difficulties for ADLs, the risk was 95% higher than in those who had preserved functional capacities (Table 2). Going to parties maintained the protective effect, associated with a 17% reduction in the risk of death (p=0.02). After the adjustment, going out of the house continued to have an important protective effect on survival, with a 39% reduction in mortality in those who went out every day of the week (p<0.001) (Table 2).

## Discussion

The effect of social relationships on the survival of the older adults was the object of the study after eight years of monitoring the older adult cohort in Bagé, RS. The median age of the older adults (78.0) at the end of the follow-up (2016/17) was similar to the life expectancy in the state of Rio Grande do Sul (77.8) and higher than that of the country (75.8). In 2010, the municipality of Bagé had an MHDI of 0.740, considered high, in which the dimension that most contributes to this reach is longevity, with an index of 0.848, followed by income (0.739) and by schooling (0.647). The site also has a political infrastructure to support the Secretariat for Older Adult Care, which promotes the integration of the areas of social assistance, health, and well-being for this population.

The article explored the functional and structural aspects of social relations^(^ 8 ^-^ 9 ^)^ and the survival analysis identified a positive association between going to parties and going out of the house with the age of death. The use of age as a measurement unit for the survival time means that the effect of the independent variables on the risk of death is naturally adjusted for the individual’s age.

Going to parties and going out of the house at the beginning of the follow-up showed the ability to predict the probability of death, with a protective effect on the survival of individuals aged 60 years old or older. Except for a trend to increase the risk with the decrease in the number of times a week in which the older adults reported going out of their houses, the findings remained after adjusting for the variables of gender, schooling, economic classification, smoking, functional difficulty, and number of residents in the home. Going to parties and going out of the house exposes individuals to various opportunities for interpersonal involvement, whether psychosocial, emotional, cognitive, cultural, recreational, or work-related^(^ 23 ^)^. The positive psychological effects of social connections are fundamental not only for the emotional and affective well-being of the older adults, but also for their health conditions, preventing morbidities^(^ 26 ^)^ and early hospitalization^(^ 27 ^)^.

The difference in mortality between men and women showed greater female longevity, corroborating national and international longitudinal studies^(^ 11 ^,^ 28 ^)^. A study carried out in Viçosa, MG, with women aged 60 or over, showed that one of the positive aspects of female old age is the expansion of social participation, with the possibility of carrying out activities hitherto limited due to responsibilities with children and household chores^(^ 29 ^)^. This explanation raised the hypothesis of an interaction between the gender of the older adult and the number of residents in the home.

However, the results showed that the effect of gender on survival can be modified by the number of residents in the home. For older adults living with two or more people, the risk of death was higher in women than in men. Brazilian women are living longer and with better living conditions due to the expansion of social security coverage, access to health services, and growth of the medical technology^(^ 30 ^)^. Family rearrangements resulting from the presence of chronic morbidities and functional difficulties may be necessary and, in the case of Brazil, in households headed by women the return of children and grandchildren to the home has been observed, when the nests are no longer empty and, although in low proportions, mothers and in-laws are also seen living in households headed by children^(^ 30 ^)^. Our results suggest that, in households with an older adult and two or more people, the older person is overburdened.

The expansion of the coverage of the Family Health Strategy (FHS) reduced hospitalizations for asthma, heart problems, and stroke, with a consequent decrease in mortality due to these conditions^(^ 31 ^-^ 32 ^)^. In addition to the FHS, policies such as the *Bolsa* *Família* (Family Grant), when effective, can explain part of the results herein found. It is possible that the effect of schooling and socioeconomic status, well-known social determinants but which in our study are not associated in isolation, has been attenuated by these and other public policies. Furthermore, it is possible that these policies explain the interaction found between schooling and socioeconomic status.

In the Bagé cohort, the effect of schooling and economic condition on the probability of death does not happen in isolation, but in interaction. The results suggest that illiterate older adults with lower economic conditions have survival mechanisms that minimize the effects of poverty, reducing social inequity. On the other hand, in the English Longitudinal Study of Ageing (ELSA) cohort, it was observed that during the first 5-year period of the study (2002-2007), 5% of the men in the richest quintile had died compared to 18% of the men in the poorest wealth quintile, with equivalent values of 3.3% and 15.6% for women. The amplitude of the socioeconomic measure in ELSA allowed verifying the effects of selective mortality^(^ 14 ^)^. The social inequalities involved in determining health situations compromise equitable healthy aging; and access policies, health promotion strategies, disease prevention, and care for the older adults with chronic conditions can minimize the effects of inequality and promote health equity^(^ 35 ^-^ 37 ^)^.

The study corroborates global findings of the association between smoking and mortality. For example, the North American cohort, with follow-up from 1993 to 2010, showed a 1.5 times higher risk in older adults who smoked compared to non-smokers^(^ 11 ^)^. The presence of multimorbidity (arterial hypertension, diabetes mellitus, cognitive deficit, depression, spinal problems, and rheumatic diseases), with the inclusion of morbidities of different etiologies, did not show any association in the risk of death in the Bagé cohort. The presence of chronic diseases in the older adult population does not necessarily mean risk, limitation or lack of autonomy in the aging process. Public policies, such as primary health care, can ensure the control and care of chronic diseases and strengthen the promotion of healthy aging, overcoming biological characteristics by offering comprehensive care under the eyes of social determination, aiming at greater autonomy and well-being of the older adult^(^ 15 ^,^ 35 ^-^ 36 ^)^.

On the other hand, the presence of disabilities can make it difficult for the older adults to go out of their houses daily and reinforces the importance of a structured support network^(^ 23 ^)^. In the Bagé cohort, the presence of limitations for basic activities of daily living tripled the mortality rate, decreased the median survival in ten years and, adjusted for the other variables in the model, practically doubled the immediate risk of death.

The presence of intergenerational support and the support of the health networks are seen as fundamental for ensuring survival in old age, especially in low-income older adults^(^ 25 ^)^. In this perspective, it is necessary to discuss strategies for healthy, successful, and equitable aging. A qualitative research study carried out with older adults in the city of Rio de Janeiro identified that social participation, conviviality and interaction, support, and family contact, in addition to carrying out leisure activities and daily tasks with autonomy and independence, are practices that promote quality life^(^ 37 ^)^.

In our study, the MCA carried out before the survival analysis showed less explanatory power for the variables on the number of visits made than for going out of the house or going to parties. The association between the frequency of going out of the house and mortality in older adults with a focus on mobility was verified in a cohort conducted in Jerusalem (1990 and 2017), and the Bagé cohort corroborates those results: greater survival for those who went out of their houses every day, regardless of social vulnerability, functional decline, physical activity, and comorbidities^(^ 23 ^)^.

The data collection time spent for the follow-up caused by the restriction of public funding can be pointed out as a limitation of the study. However, the cohort design of this study, the analysis by multiple correspondences, and the time measurement scale considering the age of death allowed us to know details of the mortality rate of the older adults in Bagé and constitute strengths of the study. On the theme of social relationships, it contributes to scientific knowledge by showing the importance of easy-to-apply questions for health professionals to identify risk markers to the detriment of using some extensive scales. The results also indicate the relevance of the role of nursing in the consolidation of longitudinal care, extrapolating social bonds and interactions in health promotion and clinical care.

## Conclusion

Strengthened social relationships play a mediating role in survival. There is some contextual factor in Bagé in favor of equity for the older adults, with statistical significance remaining for the isolated effects of schooling and socioeconomic status, and among the poorest, providing protection to the most vulnerable. Among the older adults in Bagé, living with two or more people, the risk of death for women is almost twice than that for men. Knowing the death distribution, the median age of its occurrence, and the death rates according to the exposures allowed verifying the importance of going out of the house as a risk marker. Mortality is one of the most relevant indicators for the knowledge of the health situation of a population, while the analysis by time until the occurrence of the event - survival analysis - offers more robust methods to identify the risk factors that, isolated or together, influence the pattern of mortality and can subsidize the planning of social protection measures. Articulated actions between the health services and the informal support network, including the promotion of recreational and leisure activities outside the home in healthy environments, have the potential to protect against mortality.
